# Emphasizing symbolic capital: its superior influence on the association between family socioeconomic status and adolescent subjective well-being uncovered by a large-scale multivariate network analysis

**DOI:** 10.3389/fpsyg.2024.1335595

**Published:** 2024-07-17

**Authors:** Yaozhi Wang, Wei Li, Xuerong Liu, Qianyu Zhang, Desheng Lu, Zhiyi Chen

**Affiliations:** ^1^College of Education Science, Sichuan Normal University, Chengdu, China; ^2^Experimental Research Center of Medical and Psychological Science, School of Psychology, Third Military Medical University, Chongqing, China; ^3^School of Public Administration, Chongqing University, Chongqing, China

**Keywords:** students’ subjective well-being, family socioeconomic status, adolescent students, symbolic capital, network analysis

## Abstract

**Background:**

Family socioeconomic status (FSES) serves as a significant determinant for subjective well-being. However, extant research has provided conflicting evidence on the correlation between FSES and adolescent students’ subjective well-being (SSWB).

**Methods:**

Data were collected from 12,058 adolescent students (16 years of age) by the Programme for International Student Assessment (PISA) 2018. Multivariate canonical correlation and Mantel test were utilized to investigate the specific connection between FSES and SSWB. Furthermore, a Gaussian EBICglasso graph-theoretical model was used to capture the topological properties of the FSES-SSWB network and reveal the interplay among multifarious components of FSES and SSWB.

**Results:**

FSES was positively correlated with SSWB. In the FSES-SSWB network, parental educational attainment and occupation status demonstrated the highest centrality values, thereby contributing significantly to the relationship between FSES and SSWB. However, family wealth, along with educational and cultural resources, displayed lower centrality values, signifying their weaker roles in this relationship.

**Conclusion:**

Our findings suggest that symbolic capital, rather than family affluence, exerts a dominant influence on adolescent SSWB. In other words, SSWB may not be detrimentally influenced by a deficiency in monetary resources. However, it is more susceptible to being unfavorably impacted by inferior parental educational attainment and occupational standing.

## 1 Introduction

Feelings of subjective well-being (SWB) are especially sensitive in adolescence, which is a developmental period characterized by a variety of biological, cognitive, and social changes (Christie and Viner, 2005; Hanson and Chen, 2007). Consequently, numerous efforts have been made to explore ways to enhance SWB and guide the multifaceted development of adolescents (Park, 2004; Bowers et al., 2015; Kansky et al., 2016). For instance, the United Nations Educational, Scientific, and Cultural Organization (UNESCO) has prioritized the promotion of well-being among children and adolescents in its education strategy (UNESCO, 2016). Despite this, there is cumulative evidence that SWB in young students has been consistently declining worldwide (Marquez, 2021). A recent study revealed a steady decline in the SWB of American youth since 2012 (Twenge et al., 2018). Furthermore, similar issues are emerging in European regions, with one-third of 15-year-old students living in Organization for Economic Co-operation and Development (OECD) member countries reporting low life satisfaction (OECD, 2019). This phenomenon has been observed in Asia as well, with South Korean adolescent students experiencing low SWB (Yoo and Choi, 2016). In summary, a decline in SWB among students during adolescence is prevalent across different cultures. One promising theoretical explanation attributes this global decline in SWB to the socioeconomic disadvantages faced by students during adolescence, particularly in terms of family socioeconomic status (Bradley and Corwyn, 2002). Several lines of empirical evidence support this claim by demonstrating a strong link between higher socioeconomic status and enhanced SWB among adults (Lelkes, 2006; Sacks et al., 2012; Huang et al., 2017). Despite this evidence, little is known about whether this linkage could be generalized to adolescent students.

Numerous studies have consistently demonstrated that students’ subjective well-being (SSWB) serves as a robust predictor of students’ academic performance and mental health (Bortes et al., 2021; Tsouloupas and Voulgaridou, 2021; Cárdenas et al., 2022; Blasco-Belled et al., 2024). As previously stated, unexpected declines in SSWB place students at risk of experiencing poor performance, low self-esteem and psychological issues (Hair et al., 2015; Peverill et al., 2021; Szcześniak et al., 2021). Meanwhile, the existing studies illustrated that the subjective well-being among adolescents was not optimistic, with evidence showing that 4 out of every 100,000 of them died from suicide at home or even school per year (UNICEF, 2021). Hence, researchers are proactively exploring the risk factors contributing to lower SSWB, with particular emphasis on the impact of students’ FSES on SSWB (Knies, 2012; Main, 2014; Manzoor et al., 2015; Yang et al., 2022). Typically, a student’s FSES is gauged by amalgamating their family’s wealth and cultural resources, along with their parents’ educational and occupational status (Wong et al., 2022). Recent studies indicate a positive association between family socioeconomic status (FSES) and students’ subjective well-being (SSWB), with higher FSES corresponding to increased SSWB (Wang S. et al., 2022; Wang Y. et al., 2022). However, inconsistencies exist within this area of research. For instance, an empirical study by Kim and Chung (2021) revealed no significant correlation between FSES and SSWB in adolescents (Kim and Chung, 2021). Furthermore, a separate study identified FSES as a negatively correlated factor with SSWB in adolescents (*r =* −0.270, Jurecska et al., 2012). Accordingly, the relationship between FSES and SSWB warrants systematic investigation and clearer elucidation to enhance our understanding and potentially improve SSWB.

One potential factor contributing to these inconsistent observations is the oversimplification of FSES and SSWB measurements. As previously mentioned, FSES and SSWB encompass a multitude of components, each measured heterogeneously, including wealth resources, as well as parental education level and occupation. To address these measurement-derived variations in traditional correlational models, the multivariate statistics could be a promising approach to examine these associations. Canonical correlation analysis (CCA) is a suite of multivariate statistical techniques utilized to ascertain linear relationships between variables from two distinct sets (Uurtio et al., 2018). Therefore, the utilization of CCA contributes to integrating all components of FSES and SSWB, thereby elucidating the relationship between FSES and SSWB in this study. Nonetheless, an inherent limitation of CCA is its inability to account for the intricate interplay among all elements within each set. Therefore, to compensate for this limitation, we employed network analysis following the CCA analysis. Network analysis offers a methodological approach to simultaneously depict the structure and interplay of multiple variables (Borsboom and Cramer, 2013). The unique configuration of the network enables the comprehension of intricate interrelationships among variables that traditional statistical methods cannot reveal (Jones et al., 2021). In this context, using CCA in conjunction with network analysis to delve into the intrinsic interplay between FSES and SSWB could provide primary evidence to elucidate this association in greater detail.

In the current study, we utilized CCA to elucidate the multivariate linear association between FSES and SSWB in a large-scale sample (*n* = 12,058) obtained from the Programme for International Student Assessment (PISA) 2018. This analysis separately modeled all the components of both FSES (e.g., family affluence, parental educational level, and parental occupation status) and SSWB (e.g., students’ self-efficacy, emotional experience, and sense of belonging). Subsequently, we performed a Mantel test to analyze the network-based correlation among these components. To further probe how these components interacted, we conducted a large-scale multivariate network analysis and constructed a Gaussian EBICglasso graph-theoretical model to capture the topological properties of the FSES-SSWB network. In summary, the aim of the present study was to explore the precise association between FSES and SSWB and attempt to shed light on the underlying mechanism of the FSES-SSWB relationship to promote better SSWB.

## 2 Materials and methods

### 2.1 Data and participants

In the current study, we analyzed data from PISA 2018. The Organization for Economic Co-operation and Development (OECD) organizes this international project for adolescent students every 3 years. We used the PISA 2018 dataset in the present study because it not only offered a comprehensive framework for assessing global SSWB but also provided extensive details on FSES. This study has been formally approved by the Institutional Review Board of College of Education Science in Sichuan Normal University (IRB-20230906022).

### 2.2 Measures

#### 2.2.1 Students’ subjective well-being

Subjective well-being (SWB) refers to individuals’ affective experiences and cognitive assessments concerning their lives, based on the events that occur within them (Lucas and Diener, 2008; Jebb et al., 2018). Generally, SWB encompasses five elements: positive emotion, engagement, relationships, meaning, and accomplishment (Seligman, 2011). By refining the general concepts of SWB and the engagement, perseverance, optimism, connectedness, and happiness model (EPOCH) of adolescent well-being, students’ subjective well-being (SSWB) emphasizes adolescent students’ cognitive and emotional evaluation of their school life and experiences, including self-perception, emotional experiences, interpersonal relationships, feeling of belonging, and school atmosphere (Kern et al., 2016). Based on Diener’s definition of subjective well-being and the dimensions of the EPOCH model, we incorporated 15 items into the subjective well-being questionnaire. Higher scores indicate stronger SSWB (see Table 1). The sample mean SSWB total score was 46.07 (SD = 7.35).

**Table 1:** Description of students’ subjective well-being variables.

Variable	Description
Growth mindset (GRM)	Students’ beliefs that their abilities and intelligence can develop over time.
Meaning of Life (MLI)	Students’ beliefs that their life have satisfactory significance and aspirations.
Self-efficacy (RES)	Students’ beliefs regarding their pride in achievements, their capacity to navigate complex circumstances, their competency in multitasking, and their faith in their own resilience.
Fear of failure (FFA)	Whether students worry about others’ opinions of them when they fail, whether they worry about their own abilities, and whether they doubt their future plans.
Attitudes toward competition (COM)	Whether students enjoy working in situations that involve competition from other people, whether it is important to outperform others in the task, or whether they work harder on the task compared to other people’s level of competition.
Learning goals (LGO)	Whether students’ goal is to learn to master as much of the class material as possible and to understand the content thoroughly.
Master work motivation (WMA)	Whether students are satisfied with working hard and improving their grades, whether they are sticking to tasks presented or endeavoring to master areas of potential weakness.
Student competition (COMPER)	Whether students at their school seem to value competition, whether they enjoy competing with each other, whether they feel they are always being used to compete with others.
Student cooperation (COO)/(COOPP)	Whether students advocate collaboration, whether they collaborate with each other, whether they feel that collaboration is important, and whether they feel they are encouraged to collaborate with others.
Help others (HEO)	Students’ perceptions about their reactions to bullying, their attitudes toward protecting their peers, their views on bullying, and their willingness to stand up for bullied students.
Positive feelings (AFP)	This index measures the frequency of students encountering positive emotions, encompassing happiness, liveliness, pride, joy, and cheerfulness.
Negative feelings (AFN)	This index measures the frequency of students experiencing negative emotions, including scared, miserable, afraid, and sad.
Life satisfaction (SAT)	Students’ holistic assessment of their lives.
Sense of belonging (BEL)	Students’ feelings about school relationships and integration, including their ease in making friends, their perception of being liked by others, their discomfort, feeling of alienation, and sense of loneliness at school.
Exposure to bullying (BUL)	Whether students have been bullied, threatened, excluded, or ridiculed by other students.

#### 2.2.2 Family socioeconomic status

Family socioeconomic status (FSES) is a multifaceted conceptual construct encompassing various indicators, including income, education, and occupation, which reflect the tangible and intangible resources available to family members (Bradley and Corwyn, 2002). Typically, the measurement of a student’s FSES involves synthesizing various indicators, including their family’s wealth and cultural resources, along with their parents’ educational and occupational status (Wong et al., 2022). In the current study, we utilized the FSES questionnaire which comprised a total of seven items based on the components. These items comprised two dimensions: education and occupation of parents, and familial resources, including wealth, educational and cultural resources. The sample mean FSES total score was 138.83 (SD = 41.59). Further details regarding this questionnaire can be found in Table 2.

**Table 2:** Description of family socioeconomic status variables.

Variable	Description
Mother’s education attainment (MIS)	Mother’s the highest degree or educational level
Father’s education attainment (FIS)	Father’s the highest degree or educational level
Mother’s occupational status (BMM)	Mother’s main job
Father’s occupational status (BFM)	Father’s main job
Wealth resources (WEL)	The wealth resources variable includes information about the number of bedrooms and other material items.
Educational resources (EDU)	The educational resources include information such as whether there is a room of one’s own to study, whether educational software is installed at home and the number of E-book readers.
Cultural resources (CUL)	The cultural resources include information on classic literature, books of poetry, works of art, books on art, music, or design in students’ home.

### 2.3 Data analysis

#### 2.3.1 Canonical correlation analysis (CCA)

Our study aimed to elucidate the association between FSES and SSWB, both of which were multivariate datasets. A simplistic approach seeking a univariate linear correlation and covariance between FSES and SSWB was insufficient for this study. Therefore, we employed CCA, which we felt was the best method to explore the association between FSES and SSWB. CCA is a technique that describes the linear relationship between two random variables and performs dimensionality reduction on multivariate data by maximizing the projection of variance within the same category (Hardoon et al., 2004). This study’s variables consist of two datasets: the FSES dataset ([A], *X*) and the SSWB dataset ([B], *Y*). These datasets had a sample size of *n* and a dimension of *m*, resulting in sample matrices of *X* = *n_a_* × *m* and *Y* = *n_b_* × *m*. Therefore, CCA could identify the underlying linear relationships between the two datasets by maximizing the projection of corresponding sample matrices *X* and *Y* onto projection vectors. To achieve convergence, we employed the traditional eigenvalue decomposition optimization method. This method involves taking the derivative of the projection vectors using the Lagrange theorem, optimizing the Lagrange multipliers, and obtaining the linear coefficients based on the square root of the maximum eigenvalue (Thompson, 1984).

We used IBM SPSS 27.0.1 to perform CCA on the FSES dataset (A) as the independent variable and the SSWB dataset (B) as the dependent variable in our study. First, we identified multiple pairs of linear combinations (*U_i_*, *V_i_*) from the two variable sets and analyzed the correlation coefficient *p*(*U_i_*, *V_i_*) between them. Then, we selected the canonical correlation variables with the highest correlation coefficient. The utilization of the combination’s canonical correlation coefficient could signify the correlation between the two variable sets. Finally, we revealed the specific information of the canonical variables through the application of canonical loadings.

#### 2.3.2 Network analysis

With the rapid development of graph-theoretical statistics, large-scale network analysis has enabled integrated examinations of the interplay of multivariates (Epskamp et al., 2018). A network comprises nodes and edges, where nodes denote variables and edges symbolize their connections or interactions. After the network is constructed, analyzing it with various measures and techniques facilitates the provision of quantitative centrality indicators for each node, drawing upon the unique configuration of the network, enabling the comprehension of intricate interrelationships among variables that traditional statistical methods cannot reveal (Jones et al., 2021). Consequently, to determine the underlying patterns of the relationship between FSES and SSWB, we conducted a large-scale network analysis utilizing the R program (R Development Core Team, 2014). Within the network model, each variable from FSES and SSWB was conceptualized as a node, with the relationship between two nodes depicted as an edge (Epskamp et al., 2012). Least Absolute Shrinkage and Selection Operator (LASSO) and Extended Bayesian Information Criteria (EBIC) methodologies were employed to reduce edges within the network and select pertinent tuning parameters, thereby rendering the network more sparse and facilitating interpretation (Epskamp et al., 2012). We utilized the R packages *qgraph* (Epskamp et al., 2018) and *bootnet* (Epskamp et al., 2018) to visualize the network model, where green edges signified positive relations and red edges indicated negative associations.

#### 2.3.3 Estimation of network centrality

To further elucidate the mechanism of the relationship within the FSES-SSWB network model, we quantified the significance of each node by computing the node’s expected influence (EI) using the R package *qgraph* (Epskamp et al., 2012). Computation of EI has been deemed more suitable for networks comprising both positive and negative edges, as opposed to the traditional centrality index (i.e., node strength) in previous research (Robinaugh et al., 2016). The higher the node’s expected influence, the more significant the variable was in the network model. The bridge expected influence was computed to discern bridge variables, utilizing the bridge function within the R package *networktools* (Jones, 2017). Nodes possessing higher bridge expected influence values exhibited enhanced capability in connecting one community to others, in contrast to bridges with lower expected influence values (Jones et al., 2021). To emphasize key variables, we focused our analysis on the top five variables with the highest EI values. All analyses related to R were performed using R version 4.2.3.

#### 2.3.4 Estimation of network stability

We confirmed the robustness of the results using the case-drop bootstrap procedure in the R package *bootnet* (Epskamp et al., 2018). This procedure continuously removes cases from the original sample and recalculates the centrality index (i.e., expected influence) of the nodes in the network. If the centrality indices of the nodes exhibit minimal variation after the exclusion of a subset from the dataset, the network structure is deemed stable. Correlation stability coefficients (CS-C) value signified the highest proportion of cases that could be eliminated from the sample. Generally, the CS-C value should be no less than 0.25 and ideally above 0.50 (Epskamp et al., 2018). Subsequently, we deemed the difference between two strength indices as significant if the 1,000 bootstrap 95% nonparametric confidence intervals (CIs) did not encompass “0” (Epskamp et al., 2018). This test utilized 95% CIs to ascertain whether there is a significant difference in the weight of two edges or the strength of two nodes.

## 3 Results

### 3.1 Distribution characteristics of FSESE and SSWB

**Figure 1:**
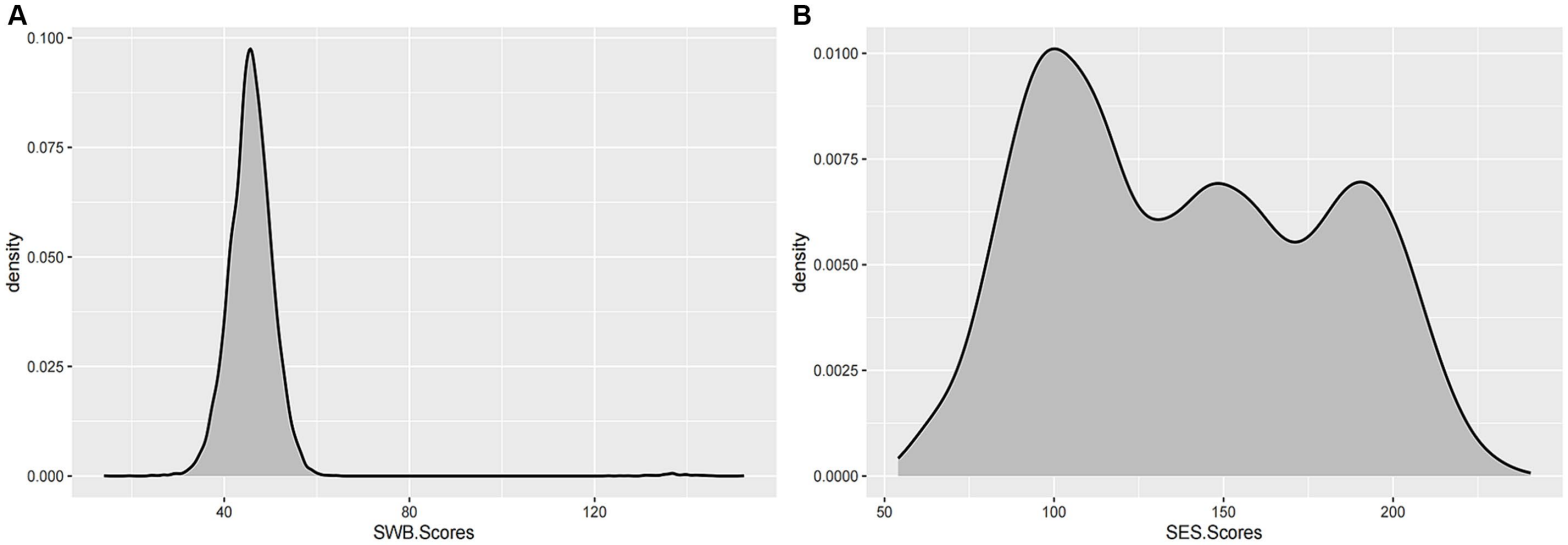
The distribution of SSWB **(A)** and FSES **(B)** for this dataset. The *x*-axis represents the total score for students’ subjective well-being and family socioeconomic status, while the *y*-axis represents the density of their scores. The gray area in the legend denotes the distribution of scores.

This study included a total of 12,058 Chinese students, with 5,775 females and 6,283 males. All participants were 16 years old. To estimate the distribution of actual total scores for SSWB, we used a k-s nonparametric distribution test. SSWB displayed a statistically significant negative skew (Figure 1A). By using this test, we further demonstrated that FSES presents a pyramid-shaped pattern with three peaks (Figure 1B). Collectively, these results highlighted the low level of SWB among adolescents. FSES scores were distributed across high, middle, and low levels.

### 3.2 Multivariate correlations between FSES and SSWB

This study conducted a CCA to examine the specific relationship between FSES and SSWB and further analyzed the extracted components of the canonical correlation variables from the CCA. Results identified a significant positive correlation between the FSES and SSWB datasets, and four pairs of significant canonical correlation variables. As shown in Table 3, significant positive correlations existed between the four pairs of canonical correlation variables: *U*_1_*V*_1_ (*r* = 0.27, *p* < 0.05), *U*_2_*V*_2_ (*r* = 0.17, *p* < 0.05), *U*_3_*V*_3_ (*r* = 0.086, *p* < 0.05), and *U*_4_*V*_4_ (*r* = 0.066, *p* < 0.05). Notably, *U*_1_*V*_1_ demonstrated the strongest correlation, with the other pairs showing weaker degrees of correlation (Supplementary material). Hence, this study restricted its interpretation of the overall relationship to this pair of canonical correlation variables (*U*_1_*V*_1_).

**Table 3:** Canonical correlation coefficients.

Canonical variable	Canonical correlation	Wilks statistic	*p* value
*U*_1_ *V*_1_	0.270	0.888	0.000
*U*_2_ *V*_2_	0.166	0.958	0.000
*U*_3_ *V*_3_	0.086	0.985	0.000
*U*_4_ *V*_4_	0.066	0.992	0.000
*U*_5_ *V*_5_	0.040	0.996	0.047
*U*_6_ *V*_6_	0.037	0.998	0.122
*U*_7_ *V*_7_	0.027	0.999	0.387

*U_i_* denotes different linear combinations of all variables of FSES, and *V_i_* represents different linear combinations of all variables of SSWB.

**Figure 2:**
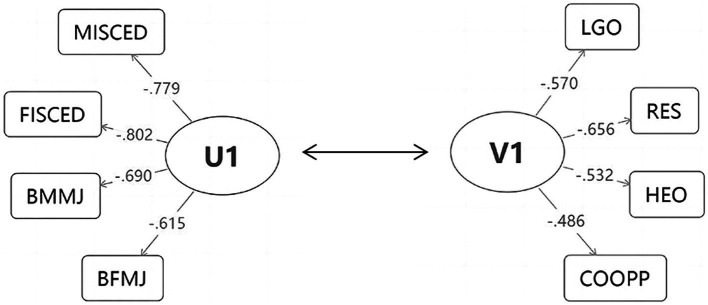
Analysis path for the results of CCA. The variables in the rectangle with the highest canonical loading among the first pair of canonical correlation variables (*U*_1_*V*_1_).

Based on the canonical loadings, we identified the key components of the first pair of canonical correlation variables (*U*_1_, *V*_1_) (Tables 4, 5). In the *U*_1_ dataset, which represented FSES, mother’s educational level (MIS, *p* < 0.05), father’s educational level (FIS, *p* < 0.05), mother’s occupation status (BMM, *p* < 0.05), and father’s occupation status (BFM, *p* < 0.05) exhibited the highest canonical loadings. Correspondingly, in the linear combination of variables in the student subjective well-being (SSWB) dataset, *V*_1_, self-efficacy (RES, *p* < 0.05), learning goals (LGO, *p* < 0.05), helping others (HEO, *p* < 0.05), and student cooperation (COOPP, *p* < 0.05) exhibited the highest canonical loading, indicating their maximal contribution to *V*_1_ (Figure 2). These results demonstrated that these variables are primary representatives of *U*_1_ and *V*_1_, and that they play a critical role in establishing the significant positive correlation observed in the CCA. Coupled with this evidence, we also observed a direct positive correlation between parents’ educational level/occupation status and students’ self-efficacy, learning goal orientation, and interpersonal interactions in school, highlighting their pivotal role in shaping SSWB.

**Table 4:** Canonical loadings of *U*_1_.

	Variable	Canonical loadings
*U*_1_	GRM	−0.313
	LGO	−0.570
	COM	−0.368
	WMA	−0.364
	RES	−0.656
	HEO	−0.532
	MLI	−0.247
	AFP	−0.230
	BEL	−0.450
	FFA	0.071
	COM	−0.345
	BUL	0.349
	COO	−0.486
	AFN	0.013

**Table 5:** Canonical loadings of *V*_1_.

	Variable	Canonical loadings
*V*_1_	WEL	−0.566
	EDU	0.473
	CUL	−0.179
	MIS	−0.779
	FIS	−0.802
	BMM	−0.690
	BFM	−0.615

### 3.3 Network-based interactions between FSES and SSWB

**Figure 3:**
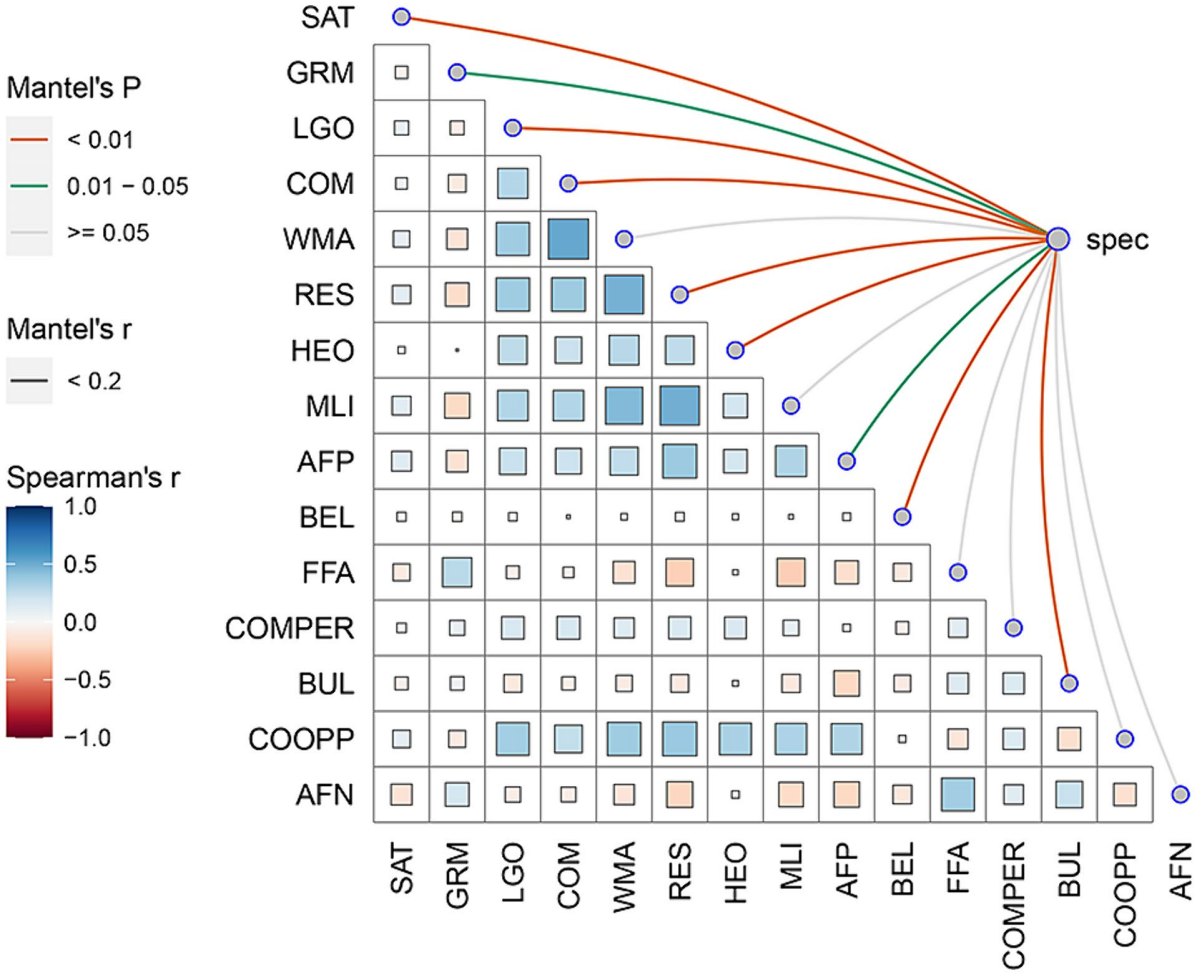
Heatmap of correlation coefficients between all variables of both FSES and SSWB. The heatmap colors represent the magnitude of the correlation coefficients. Darker colors correspond to higher correlation coefficients, indicating stronger correlations between the variables. The color scale is shown on the left side of the heatmap to indicate the range of correlation coefficients and their corresponding colors.

The foregoing CCA results provided robust evidence of a meaningful association between the two sets of variables: FSES and SSWB. To gain a comprehensive understanding of the complex interplay between FSES and SSWB, this study utilized network analysis to further investigate network-based interactions. Mantel test results revealed a statistically significant positive correlation (*r* = 0.86, *p* < 0.05, see Figure 3) between the two networks composed of all elements of FSES and SSWB.

### 3.4 FSES-SSWB network structure and centrality

**Figure 4:**
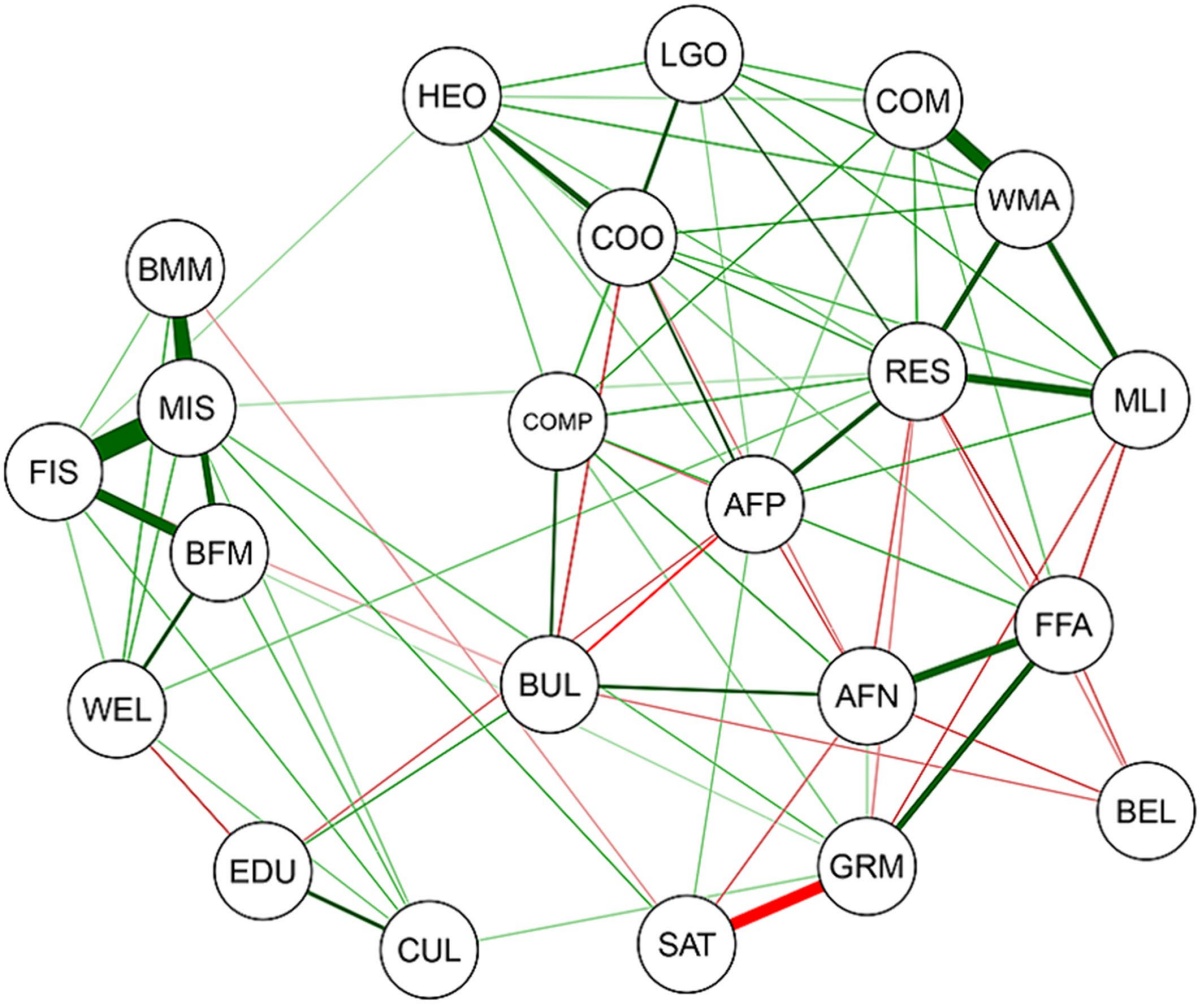
Network of SSWB and FSES. Nodes denote the variables of both FSES and SSWB. The green lines represent positive correlations. The edge thickness represents the strength of the association between nodes. GRM, growth mindset; MLI, meaning of life; RES, self-efficacy; FFA, fear of failure; COM, attitudes toward competition; LGO, learning goals; WMA, master’s work motivation; COMPER, student competition; COO, student cooperation; HEO, help others; AFP, positive feelings; AFN, negative feelings; SAT, life satisfaction; BEL, sense of belonging; BUL, exposure to bullying; FIS, father’s education attainment; MIS, mother’s education attainment; BFM, father’s occupational status; BMM, mother’s occupational status; WEL, wealth resources; EDU, educational resources; and CUL, cultural resources.

Building on the above foundation, we integrated FSES and SSWB into an FSES-SSWB network to further clarify the intricate interplay among these elements (Figure 4). Out of 231 possible edges, 85 (37%) were nonzero, indicating that the FSES-SSWB network is sparse. These results add greater theoretical validity to the network and enhance the overall interpretability and meaningfulness of the findings. In this model, we discerned the 10 most prominent edges within the FSES and SSWB communities, encompassing four edges within the FSES community and six edges within the SSWB community. The edge between FIS and MIS was strongest, followed by edges MIS-BMM, FIS-BFM, BFM-FIS, SAT-GRM, COM-WMA, MLI-RES, AFN-FFA, FFA-GRM, and HEO-COO. The within-module connectivity of FSES and SSWB was stronger than their among-module connectivity.

**Figure 5:**
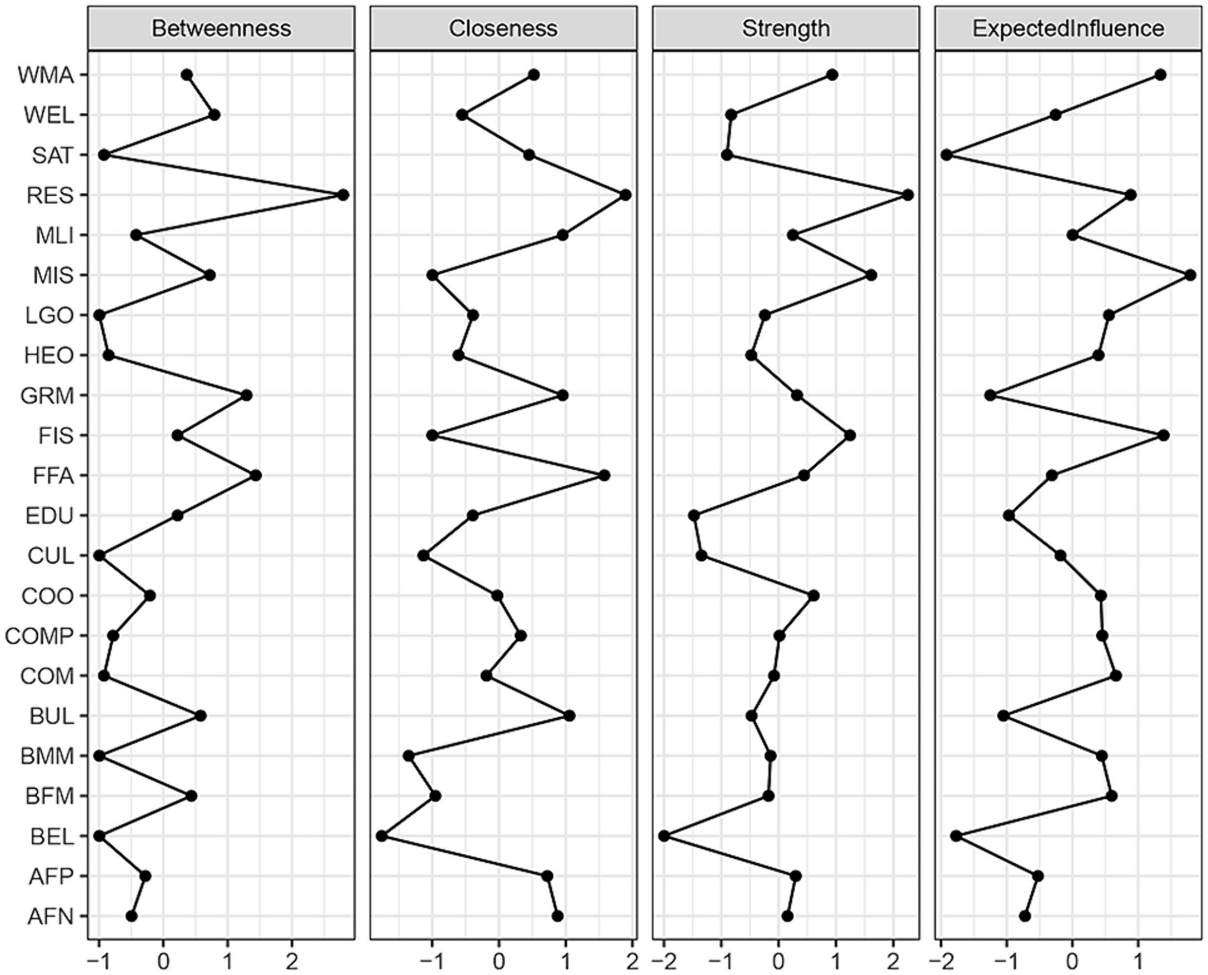
Node centrality in the network.

Figure 5 displays the expected node influences within the entire network structure. The highest expected influence value was associated with MIS, followed by FIS, WMA, and RES. These results suggested that, in terms of variance explained, MIS, FIS, WMA, and RES exert the most influence within the entire FSES-SSWB network model. This finding aligns with the results from the above CCA, indicating the paramount importance of these variables in establishing close connections within this network. Conversely, the impact of other FSES variables, such as wealth resources (WEL), cultural resources (CUL), and educational resources (EUD), was relatively marginal within the network.

**Figure 6:**
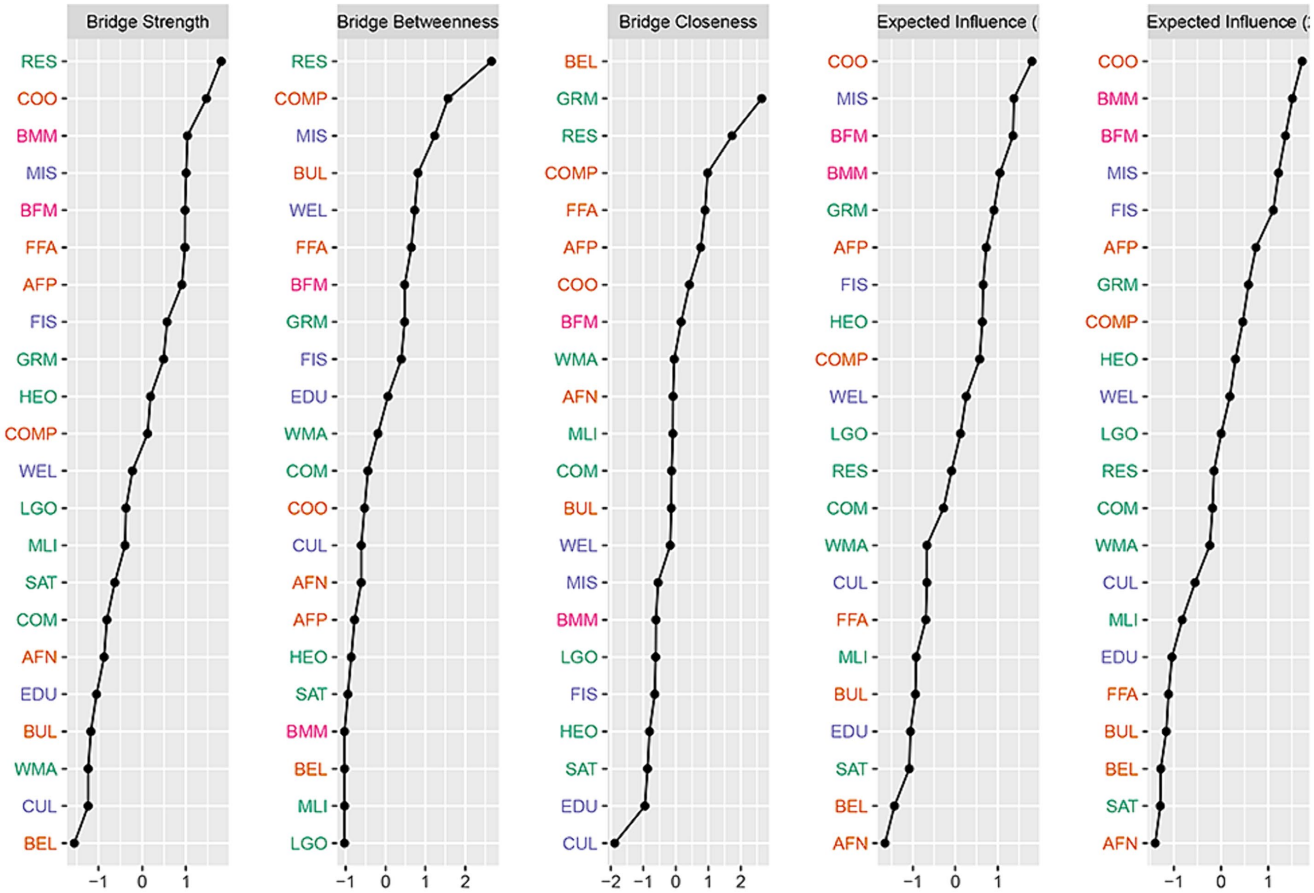
Bridge expected influence of students’ subjective well-being and family socioeconomic status.

The bridge centrality within the entire network structure is depicted in Figure 6. The variables demonstrating the highest expected influence values were students’ cooperation (COO), occupational status of mother (BMM), occupational status of father (BFM), mother’s education attainment (MIS) and father’s education attainment (FIS). These results suggest that the academic achievement and occupational status of parents have a more significant influence than material resources within FSES indicators. Specifically, these data suggest they play a crucial role in regulating and bridging collaboration or interpersonal relationships among students within schools, thereby significantly impacting SSWB.

### 3.5 Network stability

**Figure 7:**
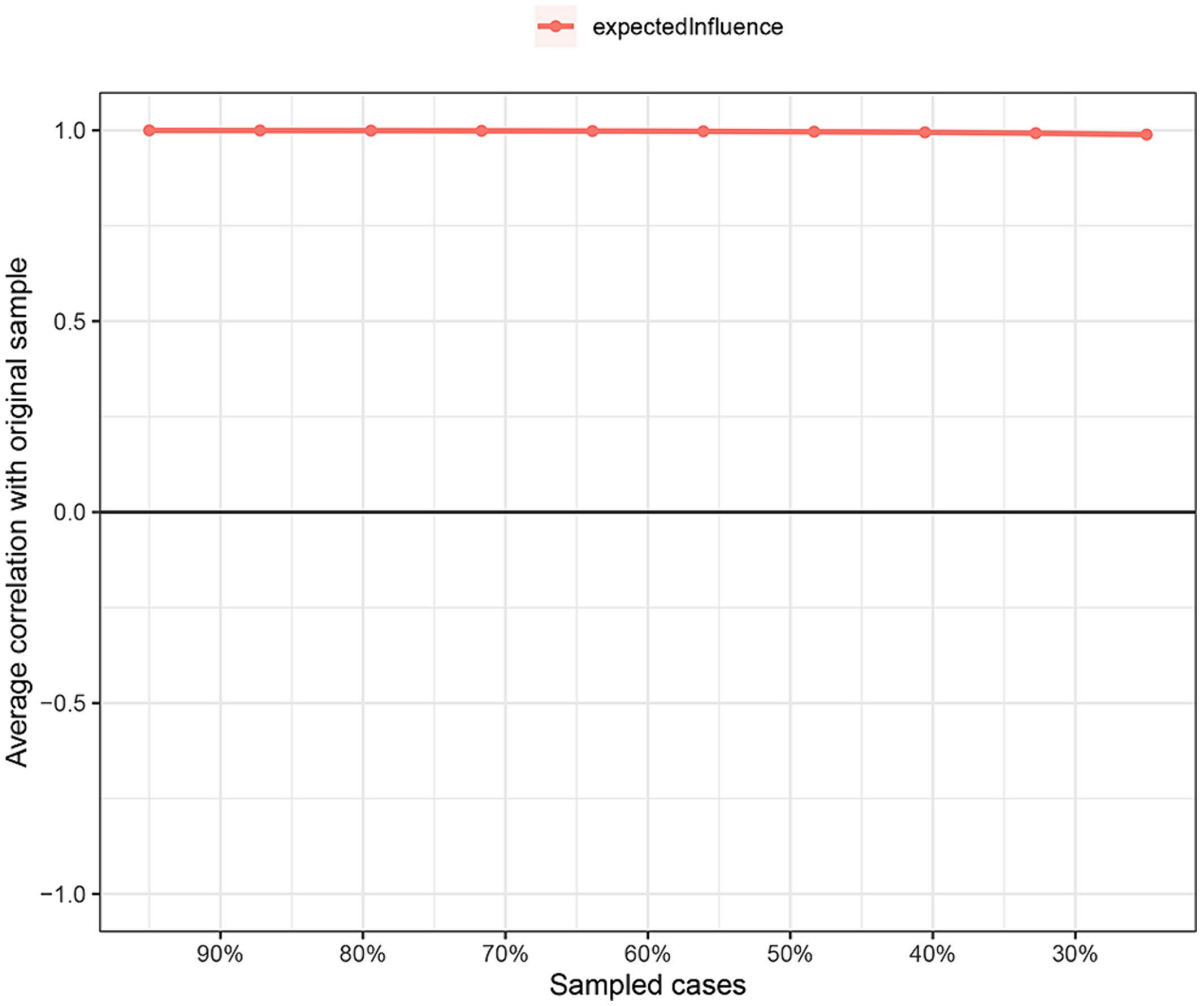
Stability of centrality indices by case dropping subset bootstrap. The *x*-axis represents the percentage of cases using the original sample. The *y*-axis displays the mean correlation between the original network’s centrality index and the re-estimated network’s index.

In terms of the stability of network analysis, the expected influence exhibited excellent stability (i.e., CS-coefficient = 0.75). This suggested that even if 75% of the sample were to be dropped, it would not result in significant alterations to the network structure (Figure 7).

## 4 Discussion

In the present study, we examined a large-scale adolescent sample to probe the relationship between FSES and SSWB, utilizing CCA and network analysis. The findings of this study collectively revealed a statistically significant positive correlation between FSES and SSWB, as well as the unique importance of symbolic capital (parental educational achievement and occupation status) in shaping SSWB. Furthermore, graph-theoretical analysis indicated that symbolic capital, represented by parental educational attainment and occupational status, exerts a greater influence on SSWB than economic and material resources. This contradicts the common argument that “money can buy well-being” (Kahneman and Deaton, 2010; Killingsworth et al., 2023). Specifically, the lack of financial resources does not necessarily result in a reduction in SWB among adolescent students. Conversely, a lower level of parental educational attainment and occupational status may likely lead to a decreased level of SWB among students. This might suggest that possessing high symbolic capital (i.e., parental educational attainment and occupational status) can potentially enhance SSWB, rather than mere financial wealth.

The results from the CCA indicated a positive linear correlation between students’ FSES and their SWB. In the CCA, we identified four pairs of significant canonical correlation variables, in which *U*_1_*V*_1_ (with *U* referring to FSES set and *V* referring to SSWB set) demonstrated the strongest correlation. Specifically, parental educational level and occupational status exhibited the highest canonical loadings in the *U*_1_ dataset, providing preliminary evidence for the significant impact of parents’ education and occupational status on the FSES-SSWB relationship. Considering the network-based interactions among the variables, we utilized a Mantel test to further investigate the FSES-SSWB correlation, thereby complementing the linear relationship established by the CCA. Mantel test results further confirmed a significant positive correlation between FSES and SSWB. Consequently, the current study provides robust evidence affirming the significant positive correlation between FSES and SSWB, supported by research that identifies FSES as a contributor to SSWB (Twenge and Campbell, 2002; Manzoor et al., 2015; Chen et al., 2016; Addae, 2020; Saunders and Brown, 2020; Treviño et al., 2021). However, this contradicts previous research suggesting a negligible or possibly inverse relationship between FSES and SSWB (Luthar, 2003; Yoo and Choi, 2016; Kim and Chung, 2021; Marquez, 2021; Wu et al., 2022). The discrepancy among studies could partly be attributed to the use of different measures for FSES and SSWB. Previous studies that used a single measure to model the FSES-SSWB relationship might have overlooked the complex interactions between the multivariate FSES and SSWB. Therefore, our study addresses the limitations of partial variable designs by employing multivariate analysis, enabling a more comprehensive and nuanced analysis. In summary, both multivariate linear and network-based analyses consistently demonstrate a significant positive correlation between FSES and SSWB.

To further understand the underlying mechanisms of the FSES-SSWB relationship, we incorporated all variables into the FSES-SSWB network. This allowed us to deconstruct the network’s topological architecture, identify the central variables of this network model, and pinpoint the highly central nodes bridging FSES and SSWB. The expected influence centrality of nodes contributed to pinpoint specific variables that rendered significant contribution to the comprehensive FSES-SSWB network. Results indicated that the centrality value was highest for parental educational attainment, classified according to the International Standard Classification of Education (ISCED) (OECD et al., 2015). This finding implies that parental educational attainment plays a pivotal role in bridging the entire FSES-SSWB network. As posited in the intergenerational transfer of socioeconomic resources model, parental educational attainment is often a key driver of the impact of other FSES factors on offspring’s SWB (Davis-Kean et al., 2021). Compared to other FSES factors, parents’ educational level plays a unique role, enabling parents to seek, identify, synthesize, and evaluate information about their children’s well-being (Davis-Kean et al., 2019), which subsequently leads to a high level of SSWB. For instance, existing literature denotes that highly educated parents are more likely to be equipped to handle stressful life situations (Reiss et al., 2019), more timely cope children’s problems to reduce the risk of their mental health problem (Horoz et al., 2022; Xiang et al., 2024), help their children develop a positive self-image (Shifrer and Pals, 2021), and earmark a larger share of their budget for family trips, school supplies, and recreational activities (Kaushal et al., 2011). These factors, in turn, contribute to increased SSWB and positive developmental outcomes for adolescents (Davis-Kean et al., 2019). Summarily, the results reveal that parents’ educational level, considered as a form of symbolic capital (Bourdieu, 2002), occupies a more central position in the FSES-SSWB network. This suggests that symbolic capital, which bestows prestige and social status, may hold more sway over SSWB than material possessions or objective economic resources.

To shed light on how the FSES-SSWB network is connected, we examined the bridge centrality within this network to enhance our comprehension of the underlying mechanisms responsible for their interaction. Within the FSES-SSWB network, the nodes with the highest five bridge centralities were “parental educational attainment,” “parents’ occupation status,” and “student cooperation.” The classification of parents’ occupation status was based on the International Standard Classification of Occupations (ISCO 08) (OIT, 2012). The term “student cooperation” refers to students’ perception of the cooperative atmosphere among their peers, reflecting their interpersonal relationships at school. The findings suggest that parents’ educational level and occupational status have stronger associations with components of SSWB, particularly student cooperation, than other FSES indicators, thereby establishing a connection between FSES and SSWB. As previous youth development research has indicated, adolescence is a crucial developmental period where peer relationships become a priority (Zhou et al., 2023). Peer relationships become more prominent during adolescence as they place greater importance on the expectations and acceptance of their peers (Xu et al., 2022). Adolescents’ focus on cooperation and interpersonal relationships typically increases during this period (Lee et al., 2022). Furthermore, cooperative interactions with peers or active peer relationships can lead to improved psychological health and SSWB (Szcześniak et al., 2022). According to Bourdieu’s Theory of Symbolic Domination, parents’ educational level and occupational status constitute a form of symbolic capital, associated with prestige and social status (Boghian, 2013). Consequently, individuals often prioritize the pursuit, perception, and even reverence of social status over the acquisition of material resources like money or income (Wang et al., 2023). Moreover, schools may favor parents who possess rich social and cultural experiences, often associated with intellectuals and social elites (Kim et al., 2023). Therefore, students from families with higher parental education and occupational status levels may receive more attention from teachers and establish better teacher–student relationships (Lareau, 1987; Tan et al., 2020), which could ultimately enhance their SWB (Baker et al., 2008; Fang et al., 2023). These previous findings suggest that parents’ educational level and occupational status are more influential than tangible possessions in shaping and bridging the relationship between FSES and SSWB.

In contrast, a hierarchical structure exists in adolescent social ecology. Adolescents can categorize or rank themselves or their peers based on sociodemographic status, power, or prestige (Rubin et al., 2006; Pattiselanno et al., 2015; Grapsas et al., 2021; Tuominen and Tikkanen, 2023). Adolescents may be more sensitive to, and influenced by, parental educational attainment and occupational status, as these factors reflect position and prestige. As indicated by previous studies, parents with a low income but high educational attainment or occupational status have a stronger influence on their children’s SWB compared to low-income parents with less education (Chen et al., 2002; Davis-Kean, 2005; Tighe and Davis-Kean, 2019). In conclusion, we deduce that parents’ educational level and occupational status are more likely to predict and influence SSWB compared to material wealth.

To sum up, the current study highlights the pivotal role of symbolic capital in the network of FSES and SSWB, which has several implications for parent, youth organization and school. First, for parents, the finding encourages parents to actively pursue educational opportunities, not just for their own occupational advancement, but also for the indirect benefits on their children (Zhang, 2021). Furthermore, as we discussed above, such symbolic capital may function as the ability to seek, identify, synthesize, and evaluate information about their children’s well-being. Therefore, parents should proactively engage in their children’s educational activities, both in terms of quantity and quality, which may potentially mitigate the adverse effects of lower FSES on adolescent development and SSWB (Doi et al., 2020; Li and Guo, 2023). Second, as limited by financial resources, those parents with low FSES may not be able to afford the cost of further education. Therefore, youth organizations should pay more attention to the pivotal role of parental educational attainment and offer educational resources in collaboration with the government, which may yield more substantial benefits than those focusing solely on economic resources (Ge, 2020; Zhang, 2021; Jin, 2022). Third, such symbolic capital is also associated with prestige and social status, leading to decreased SSWB for adolescent students with lower FSES, due to school’s differential and unequal treatment (Montoro et al., 2021). Therefore, it is important for schools to strive to avoid disclosing the information regarding students’ FSES and develop related policies. Furthermore, caregivers in the school (e.g., teachers) should also give fair attention to all students, irrespective of their FSES, to mitigate the adverse impacts of low FSES on their self-esteem, and foster positive, caring, and supportive environments and promote mutual assistance among students in learning and life (Tan et al., 2020).

## 5 Limitations and future directions

Despite novel findings in the current study, several limitations warrant caution. First, because the sample for this study was exclusively drawn from China, future research may explore the relationship between FSES and SSWB in a cross-cultural context. Second, using cross-sectional data cannot establish a causal inference for the interplay between FSES and SSWB. Future research could utilize longitudinal studies to better establish the causal relationship between FSES and SSWB. Third, FSES data distribution in this study was relatively skewed. However, this skewed distribution aligns with the real-world situation of wealth distribution in China, which follows a pyramid shape (Piketty et al., 2019). Therefore, future studies should include more balanced data samples from multiple cultures and regions to provide a more complete picture of the interrelationship between FSES and SSWB. Last, we narrowed the scope of this study to the adolescents aged 16, limited by the sample collection of PISA. Hence, examining the relationship between FSES and SSWB across adolescents of all ages could strengthen the reliability and generalizability of the findings among adolescent students.

## 6 Conclusion

The current study sheds light on the complicated network-based association between FSES and SSWB. The findings underscored that parents’ academic achievement and occupational status are more important than material wealth (e.g., money) for SWB among adolescents. In other words, material abundance does not necessarily mean a high level of SWB; however, if parents have a low level of education attainment and occupation status, SSWB is more likely to be lower. Furthermore, students’ peer relationships also play a key role in their SWB. Overall, this study provides evidence of a positive correlation between FSES and adolescents’ SWB in school, primarily attributed to superior parental educational attainment and occupational status, rather than material wealth per se.

## Data availability statement

Publicly available datasets were analyzed in this study. This data can be found here: https://osf.io/FRQ7Z at: DOI: 10.17605/OSF.IO/FRQ7Z.

## Author contributions

YW: Formal analysis, Writing – original draft, Writing – review & editing. WL: Formal analysis, Writing – original draft, Writing – review & editing. XL: Writing – review & editing, Validation. QZ: Writing – review & editing, Validation. DL: Writing – review & editing, Conceptualization, Funding acquisition. ZC: Writing – review & editing, Conceptualization, Funding acquisition.
