# Supplementary material for: Emphasizing symbolic capital: its superior influence on the association between family socioeconomic status and adolescent subjective well-being uncovered by a large-scale multivariate network analysis
Source: Front Psychol. 2024 Jul 17;15:1335595. doi: 10.3389/fpsyg.2024.1335595 (PMC11288923; doi:10.3389/fpsyg.2024.1335595)
Supplement: Supplementary file 1 [file Table_1.DOCX]

Supplementary Material

Emphasizing Symbolic Capital: Its Superior Influence on the Association Between Family Socioeconomic Status and Adolescent Subjective Well-Being Uncovered by a Large-Scale Multivariate Network Analysis

Yaozhi Wang^1^† , Wei Li^2^†, Xuerong Liu^2^, Qianyu Zhang^3^, Desheng Lu^1^*, Zhiyi Chen^2^*

^1^College of Education Science, Sichuan Normal University, Chengdu, China

^2^Experimental Research Center of Medical and Psychological Science, School of Psychology, Third Military Medical University, Chongqing, China

^3^School of Public Administration, ChongQing University, Chongqing, China

*** Correspondence:**

**Desheng Lu**

deshenglu@sicnu.edu.cn

**Zhiyi Chen**
[chenzhiyi@tmmu.edu.cn](mailto:chenzhiyi@tmmu.edu.cn)

†These authors share first authorship.

# Supplementary Figures and Tables

## Supplementary Tables

Table 1. Canonical Loadings of U_2_

|  | Variable | Canonical loadings |
| --- | --- | --- |
| U_2_ | GRM | -0.517 |
|  | LGO | 0.169 |
|  | COM | 0.190 |
|  | WMA | 0.374 |
|  | RES | 0.198 |
|  | HEO | 0.020 |
|  | MLI | 0.318 |
|  | AFP | 0.708 |
|  | BEL | 0.141 |
|  | FFA | -0.030 |
|  | COM | -0.199 |
|  | BUL | -0.542 |
|  | COO | 0.303 |
|  | AFN | -0.248 |

Table 2. Canonical Loadings of V_2_

|  | Variable | Canonical loadings |
| --- | --- | --- |
| V_2_ | WEL | 0.095 |
|  | EDU | -0.732 |
|  | CUL | -0.656 |
|  | MIS | -0.354 |
|  | FIS | -0.331 |
|  | BMM | -0.282 |
|  | BFM | -0.303 |

Table 3. Canonical Loadings of U_3_

|  | Variable | Canonical loadings |
| --- | --- | --- |
| U_3_ | GRM | -0.279 |
|  | LGO | -0.045 |
|  | COM | 0.156 |
|  | WMA | 0.277 |
|  | RES | 0.598 |
|  | HEO | -0.275 |
|  | MLI | 0.511 |
|  | AFP | 0.014 |
|  | BEL | -0.192 |
|  | FFA | -0.081 |
|  | COM | 0.126 |
|  | BUL | 0.322 |
|  | COO | 0.219 |
|  | AFN | 0.106 |

Table 4. Canonical Loadings of V_3_

|  | Variable | Canonical loadings |
| --- | --- | --- |
| V_3_ | WEL | 0.555 |
|  | EDU | 0.441 |
|  | CUL | -0.403 |
|  | MIS | 0.220 |
|  | FIS | -0.002 |
|  | BMM | 0.590 |
|  | BFM | -0.027 |

Table 5. Canonical Loadings of U_4_

|  | Variable | Canonical loadings |
| --- | --- | --- |
| U_4_ | GRM | -0.010 |
|  | LGO | 0.129 |
|  | COM | -0.252 |
|  | WMA | 0.086 |
|  | RES | -0.021 |
|  | HEO | 0.122 |
|  | MLI | 0.252 |
|  | AFP | -0.324 |
|  | BEL | 0.112 |
|  | FFA | -0.057 |
|  | COM | -0.476 |
|  | BUL | -0.155 |
|  | COO | 0.373 |
|  | AFN | -0.392 |

Table 6. Canonical Loadings of V_4_

|  | Variable | Canonical loadings |
| --- | --- | --- |
| V_4_ | WEL | -0.532 |
|  | EDU | -0.021 |
|  | CUL | -0.362 |
|  | MIS | 0.399 |
|  | FIS | 0.207 |
|  | BMM | -0.100 |
|  | BFM | -0.284 |

## Supplementary Figures


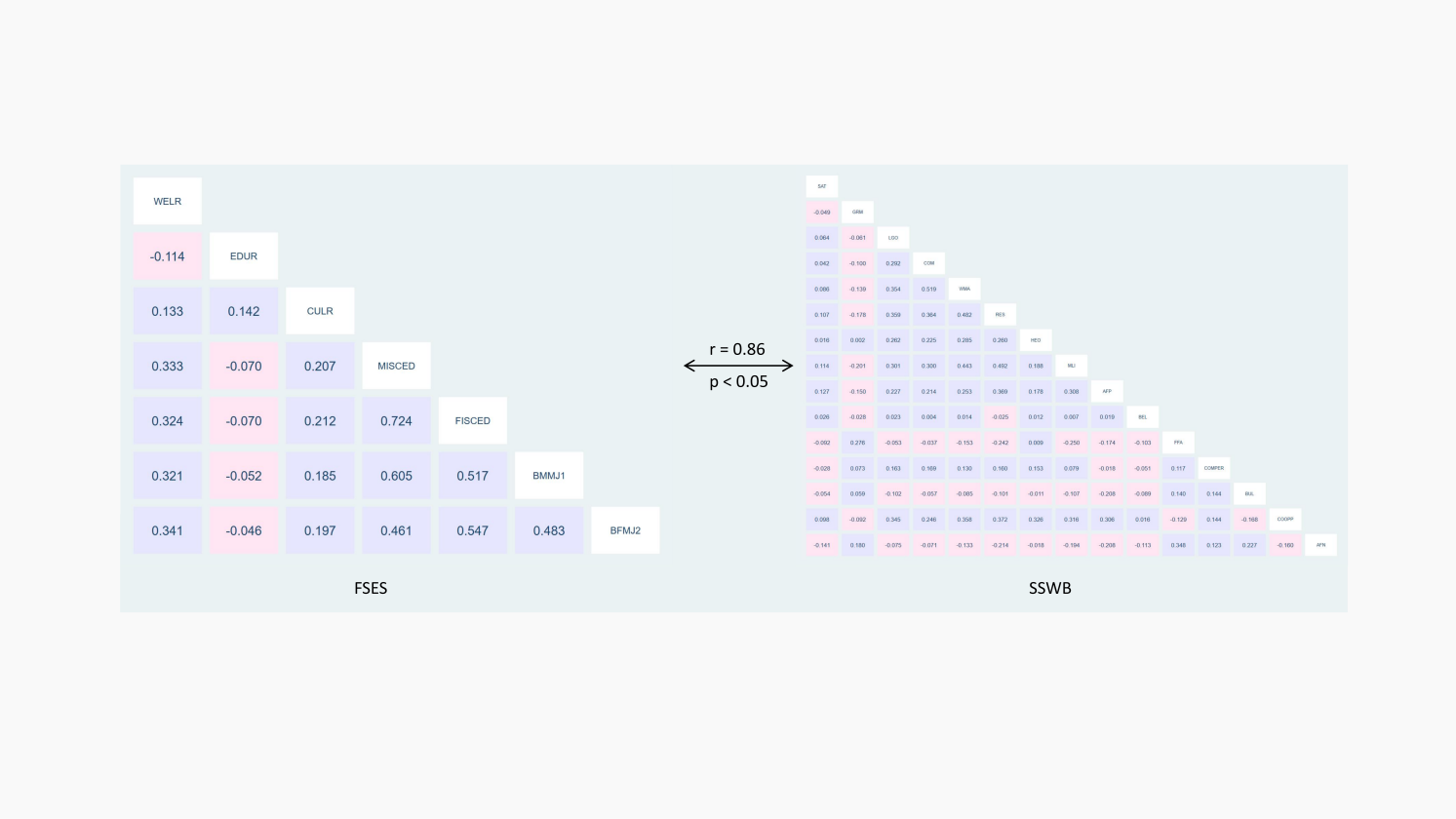


**Supplementary Figure 1.** The figure, generated using STATA, illustrates the correlation between the two networks composed of all the elements of FSES and SSWB. The purple boxes represent positive correlations. The pink boxes represent negative correlations.
